# Letter in response to the case report “Topical ruxolitinib in the treatment of refractory facial seborrheic dermatitis”

**DOI:** 10.1016/j.jdcr.2023.09.038

**Published:** 2023-10-15

**Authors:** Meron Teklu, Hye Jin Chung

**Affiliations:** a Department of Dermatology, Harvard Medical School, Boston, Massachusetts; b Harvard Combined Dermatology Residency, Boston, Massachusetts; c Department of Dermatology, Beth Israel Deaconess Medical Center, Boston, Massachusetts

**Keywords:** seborrheic dermatitis, ruxolitinib

*To the Editor:* We read with interest a recent case report by Pope et al^1^ of a patient who had complete resolution of his seborrheic dermatitis (SD) and partial resolution of rosacea after 2 weeks of treatment with ruxolitinib cream. We are enthusiastic to share our experience of refractory SD successfully treated with topical ruxolitinib 1.5% cream.

A 27-year-old man presented with pruritus for 7 years and scale of the face, ears, scalp, and chest. Physical examination revealed erythematous scaly papules and plaques on the nose, nasolabial fold, medial cheeks, and chest. He was clinically diagnosed with SD and initially treated with ketoconazole cream 2 times a day for 2 months, followed by zinc pyrithione shampoo 3 times a week for maintenance. He presented again to clinic several years later with continued pruritic rash and was started on betamethasone cream twice a day for 1 week per month, followed by ketoconazole cream daily, without much improvement and subsequent spread of his rash to the bilateral cheeks. He was then started on oral fluconazole 200 mg weekly, azelaic acid 15% gel 2 times a day, and pimecrolimus cream 2 times a day. He experienced some initial improvement but subsequently worsened with re-emergence of his rash and pruritus over the next several months. For a possible component of demodex folliculitis, he was also given oral ivermectin 21 mg weekly for 4 weeks in addition to topical hydrocortisone 2.5% for the face, triamcinolone 0.1% for the chest, and ciclopirox shampoo. Despite these interventions, he continued to have persistent rash and pruritus (Figs 1, *A* and 2, *A*). Given his lack of improvement with multiple topical and oral regimens, a punch biopsy was performed on the chest, which showed epidermal hyperplasia with mild spongiosis, perifollicular neutrophilic parakeratosis, and superficial perivascular lymphocytic inflammation, consistent with SD. Given that biopsy prove extremely refractory SD, he was taken off all medications and started on ruxolitinib 1.5% cream, which he used every other day for 4 weeks. He noticed significant improvement of his rash and pruritus in the first week, and by week 4, he had near complete resolution (Figs 1, *B* and 2, *B*).

**Fig 1:**
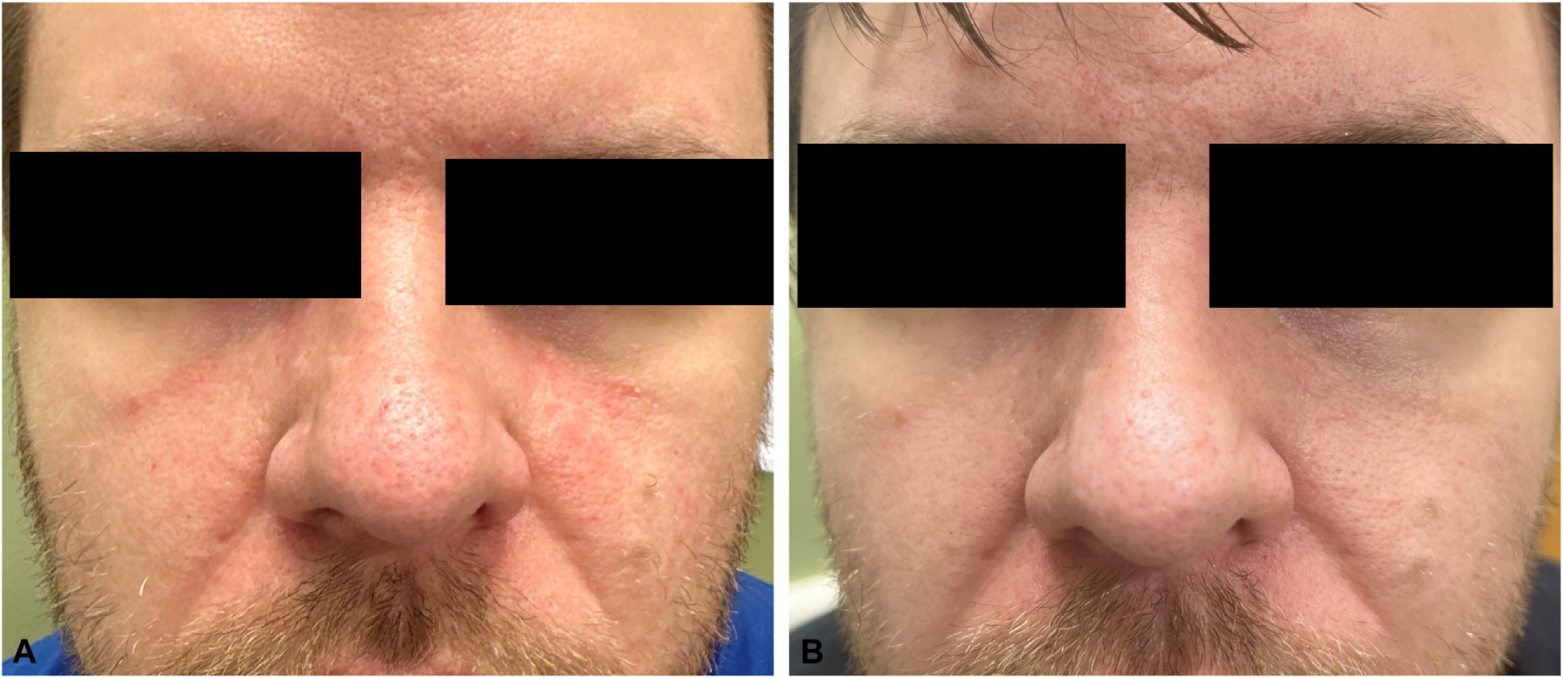
Seborrheic dermatitis of the face (**A**) before and (**B**) after ruxolitinib cream 1.5%.

**Fig 2:**
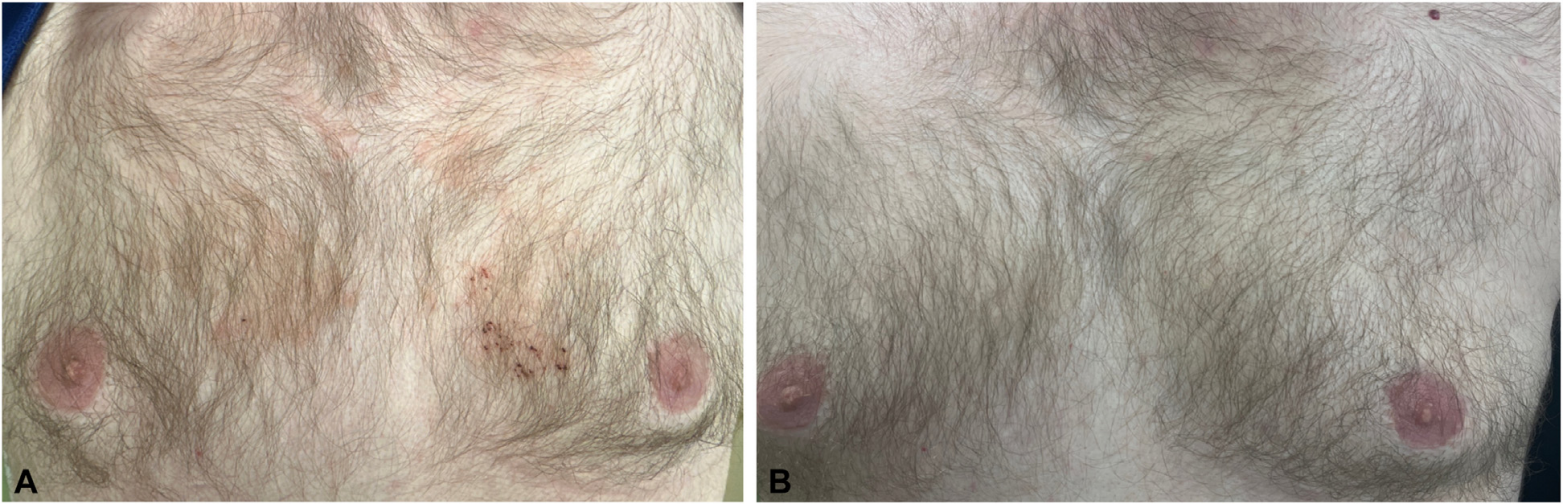
Seborrheic dermatitis of the chest (**A**) before and (**B**) after ruxolitinib cream 1.5%.

The current mainstay of treatment for SD involves medicated shampoos, topical antifungals, and topical corticosteroids. Ruxolitinib is a selective Janus kinase (JAK)1/2 inhibitor whose topical formulation (ruxolitinib 1.5% cream) is approved for use in atopic dermatitis and nonsegmental vitiligo.^2^^,^^3^ JAK inhibitors disrupt cytokine and growth factor signaling pathways, generally leading to a decrease in the inflammatory response.^4^ A small previous study has shown that punch biopsy lesions from SD show an increase in various cytokines, including interleukin 4, when compared with healthy volunteers.^5^ In mice models of dermatitis, ruxolitinib cream use resulted in downregulation of proinflammatory genes, including the interleukin 4 receptor α chain.^6^ This may offer a mechanistic hypotesis for why topical ruxolitinib may be beneficial in SD in a similar manner to other inflammatory conditions, such as atopic dermatitis. We hope that these reports add to the body of evidence suggesting that topical JAK inhibitors may play a broader role in the treatment of common dermatologic conditions.

## References

[1] Pope E., Kowalski E., Tausk F. (2022). Topical ruxolitinib in the treatment of refractory facial seborrheic dermatitis. JAAD Case Rep.

[2] Rosmarin D., Passeron T., Pandya A.G. (2022). Two phase 3, randomized, controlled trials of ruxolitinib cream for vitiligo. N Engl J Med.

[3] Papp K., Szepietowski J.C., Kircik L. (2021). Efficacy and safety of ruxolitinib cream for the treatment of atopic dermatitis: Results from 2 phase 3, randomized, double-blind studies. J Am Acad Dermatol.

[4] Tanaka Y., Luo Y., O’Shea J.J., Nakayamada S. (2022). Janus kinase-targeting therapies in rheumatology: a mechanisms-based approach. Nat Rev Rheumatol.

[5] Faergemann J., Bergbrant I., Dohsé M., Scott A., Westgate G. (2001). Seborrhoeic dermatitis and Pityrosporum (Malassezia) folliculitis: characterization of inflammatory cells and mediators in the skin by immunohistochemistry. Br J Dermatol.

[6] Scuron M.D., Fay B.L., Connell A.J., Peel M.T., Smith P.A. (2020). Ruxolitinib cream has dual efficacy on pruritus and inflammation in experimental dermatitis. Front Immunol.

